# Thermal Pharyngeal Injury Resulting From Vaping: A Case Report

**DOI:** 10.7759/cureus.61718

**Published:** 2024-06-05

**Authors:** Samantha Brophy, Jackie Combs, Julia Hutchison

**Affiliations:** 1 Emergency Medicine, California Northstate University College of Medicine, Elk Grove, USA; 2 Emergency Medicine, Kaiser Permanente Central Valley, Modesto, USA

**Keywords:** e-smoking, vaping, e-cigarettes, electronic cigarettes, burn wounds, vaping-related lung injury, airway edema, thermal burn

## Abstract

Thermal injury to the pharyngeal structures is uncommon, and clinicians generally attribute these injuries to consuming hot foods or liquids. While thermal injuries have been reported with the ingestion of hot substances, thermal injuries from vape pens have not been widely described in the literature. We present a case of a 35-year-old male who presented to the emergency department (ED) with oropharyngeal burns after utilizing a vape pen that malfunctioned. The patient had visible burns on his uvula, as well as on the soft and hard palate. Additionally, he had symptoms of difficulty swallowing and a hoarse voice, which raised concerns about a possible deeper airway or lung injury. The patient required a flexible nasopharyngolaryngoscopy by a head and neck surgeon, which revealed mild edema and erythema of the epiglottis and the arytenoids. The patient was admitted to the hospital overnight for observation and treatment with analgesia and dexamethasone. The following morning, the patient’s symptoms had improved. The repeat nasopharyngolaryngoscopy showed improvement in the swelling of the epiglottis and arytenoids, and the patient was deemed stable for discharge. This case brings attention to the variety of injuries possible from e-cigarette use and the importance of prompt management of oropharyngeal thermal injuries.

## Introduction

Electronic nicotine delivery systems, more commonly known as e-cigarettes or vapes, continue to grow in popularity despite the growing literature on the risks to users. Injuries including thermal burns, e-cigarette or vaping product use-associated lung injury (EVALI), seizures, nicotine toxicity, and trauma from exploding devices have been reported [1]. E-cigarettes and vapes share a similar design and use batteries to heat and vaporize liquid substances such as nicotine or tetrahydrocannabinol, which can then be inhaled. The batteries are one of the most dangerous parts of these devices, as they are capable of exploding and causing penetrating trauma and thermal burns [1]. The devices have also been reported to leak hot liquid, which can cause burns [1]. Thermal burns from e-cigarette malfunction have been reported as impacting a variety of anatomic locations, with only 33% being isolated to the face (including the lips and tongue) [1]. Thermal injuries to the throat are uncommon and mostly occur in young children due to the accidental ingestion of hot fluids and foods. In adults, rare cases have been reported, often in patients with preexisting cognitive disorders [2,3]. Here, we present a case of pharyngeal thermal injury secondary to e-cigarette use.

## Case presentation

A 35-year-old male with no significant past medical history presented to the emergency department (ED) for evaluation of a throat injury. The patient stated he was vaping nicotine the night prior, and when he inhaled deeply from his vape pen, he experienced an immediate, intense burning pain in the back of his throat. He later developed throat swelling, coughing, difficulty swallowing, and a hoarse voice. He had no history of similar responses to vaping prior to this episode, no known allergies, and no recent sick contacts.

**Figure 1:**
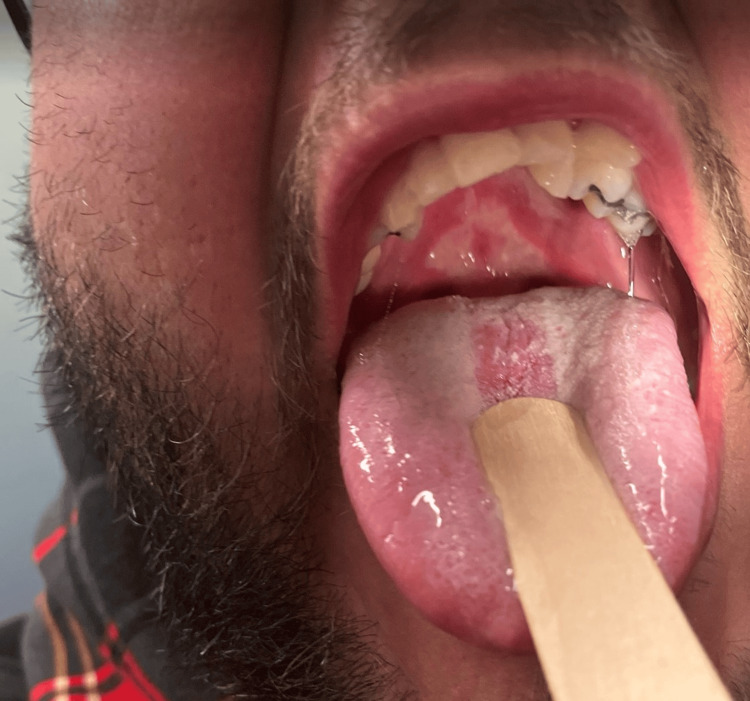
This photograph demonstrates the erythema, edema, and burn injury to the hard and soft palate.

During the examination, the patient was tachycardic and mildly tachypneic, with other vital signs stable. The ED physician noted erythema, edema, and burns on the soft palate, hard palate, and uvula (Figure 1). The neck was tender to palpation midline and along the right side. The patient had no trismus or drooling, but he was spitting up saliva. There was no submandibular or submental swelling. There was no stridor and no tracheal deviation.

During his course in the ED, he received ketorolac (15 mg) intravenously, dexamethasone (12 mg) intravenously, and 2.25% nebulized racemic epinephrine solution (0.5 mL). The chest radiograph was unremarkable. The neck radiographs showed normal bony structures, no free air, and no narrowing of the airway, but the radiologist noted prevertebral soft tissue thickening measuring up to 2.8 cm at the level of the fifth cervical vertebrae. Given the concern for airway edema and deeper burns, the ED physician consulted the head and neck surgeon on call who performed flexible nasopharyngolaryngoscopy at the bedside, which revealed erythema and edema of the epiglottis and arytenoids from the burn injury. The vocal cords were easily visible, mobile, and symmetric, with no lesions. The head and neck surgeon did not expect worsening of his upper airway edema as this typically peaks at 24 hours. However, there was still concern for the development of a lower pulmonary injury, such as pneumonia or acute respiratory distress syndrome (ARDS), which can present in a delayed fashion after an inhalation injury. She recommended dexamethasone (10 mg) intravenously every six hours and admission to the hospital for overnight observation.

On re-examination the next morning, the patient reported feeling better, with a resolving cough and improved oral intake of clear liquids. The physical exam noted a raspy voice with the ability to communicate, absence of stridor, a hard palate with shallow ulcerations, a soft palate and uvula with scattered shallow ulcers, epiglottis with improved edema and erythema, and a mildly erythematous right arytenoid. Secretions had improved, the remainder of the examination was normal, and the patient was stable enough to be discharged home. Before discharge, the patient was counseled on the risks of e-cigarette use, and cessation was recommended. The patient had a telephone appointment with his primary care physician six days after discharge. The patient reported that he was doing well and that he was no longer vaping.

## Discussion

E-cigarette use is known to be capable of causing both thermal and chemical burns. Recent literature primarily focuses on EVALI and other pulmonary pathologies that have become increasingly prevalent in teens and young adults since the increase in popularity of vaping over the last 10 years. EVALI is a respiratory illness that causes damage to the pulmonary alveoli and the lower respiratory tract [4]. The severity of the disease can range from mild pneumonia to life-threatening ARDS [4]. The chemical burns in EVALI are currently believed to be associated with vitamin E acetate. Vitamin E is an oily condensing agent that can be added to the tetrahydrocannabinol vaping liquid [4]. Vitamin E has been found in the lung tissue of all studied cases of EVALI, and the CDC has identified vitamin E as a potential cause of EVALI [4]. Vitamin E is capable of staying in the pulmonary tissue for a prolonged period, and EVALI can develop days to weeks after the initial exposure [4]. A patient who develops EVALI might experience diminished oxygen saturation, shortness of breath, diminished breath sounds, or signs of consolidation on radiography [5]. Other signs of a complication can include a new-onset cough, fever, chills, rales on auscultation, tachypnea, and pleuritic chest pain. The manifestations of the pulmonary complications are vast, and these are just some of the clinical signs to be aware of when reevaluating a patient with a vaping injury.

In contrast, thermal injury secondary to e-cigarette use is more associated with device malfunction, explosion, or leaking of hot fluid and impacts a wide range of anatomic structures, with only 33% being isolated to the face and oral cavity. Thermal burns in the oral cavity and pharynx are diagnosed by clinicians through a physical exam, demonstrating characteristic features including erythema, edema, blisters or bulla, and pain following a history of consumption of hot liquids or solids, inhalation of hot vapors, or exposure to corrosive substances or electricity [6].

The management of such burns depends on their severity. In minor cases, cooling the affected area with water and maintaining normal oral hygiene with the addition of saline rinses may be sufficient [7,8]. Alcohol-based mouthwashes should be avoided until oral lesions have resolved. Vaseline and topical antibiotic ointment can be applied in cases of lip involvement [7]. In more severe cases with pharyngeal involvement, monitoring the airway for patency is essential, and endoscopy and/or intubation may be necessary to maintain airway access [7,8]. Radiographs of the chest and neck can provide additional information regarding swelling in the soft tissues of the neck and pulmonary complications [8].

## Conclusions

We are seeing a larger population, ranging from long-time smokers to never-smokers, and even children and young teens, accessing e-cigarettes and vape pens. We expect that the variety and number of vaping-related injuries will increase as the user base continues to increase. Clinicians must familiarize themselves with the variety of chemical, thermal, and traumatic injuries that can be caused by vaping. Recognizing the possible complications can ensure prompt monitoring and improved outcomes. Educating patients about the risks associated with vaping can benefit them in the long term, with a focus on preventative medicine. Expanding our role as physicians into one that includes tackling potential social issues, such as the marketing of these devices to children, can help long-term patient outcomes.
